# Physical Diagnosis

**Published:** 1895-11

**Authors:** Jacob Helmer

**Affiliations:** Scranton, Pa.


### PHYSICAL DIAGNOSIS.

BY JACOB HELMER, D.V.S.,
SCRANTON, PA.

(Concluded from page 646.)

Having now considered the various methods of physical examination and described the physical signs afforded by them, let us give attention to a brief description of the morbid anatomy of the most common pulmonary disease with the physical signs found connected with it. Then we shall see clearly how to make a differential diagnosis of these conditions.

*Pneumonia.* This disease is divided into three classes: croupous, catarrhal, and interstitial. Croupous pneumonia is the kind commonly met. It is an acute inflammation of the vesicular structure of the lung, followed by temporary blocking of the air-cells with exudate and exclusion of air. In the first stage there is an increased amount of blood in the capillary vessels. This causes pressure on the air-cells and diminishes their calibre; subsequently comes dryness, then secretion of plastic material blocks the air-cells. The tissue becomes the color of liver, being engorged with bloody sero-fibrinous exudate. The third stage, or that of resolution, is marked by the material becoming milky white; there is now again some air in the cells. Absorption goes forward. The physical signs of croupous pneumonia in the first stage appear in about twenty-four hours. If the disease is anterior or central it may take two or three days. Inspection notes diminished movement of chest-wall on affected side. On both sides it is double pneumonia; also the increased use of the abdominal muscles. Percussion in first stage gives some dulness over diseased part. Central pneumonia may not yield dulness until advanced to the second stage. Auscultation in first stage is not satisfactory until exudation has taken place. Then we hear the crepitant râle. It lasts but a few hours. Inspection in second stage shows the wall over the affected lung to be more quiet than in the first stage. Movement of the normal side increased. Percussion yields dulness over the diseased part of the lung, while normal portions yield increased resonance. Auscultation in the second stage yields no vesicular murmur over the affected portion. The crepitant râle has disappeared and bronchial breathing taken its place. The more condensed the lung-tissue the plainer the bronchial breathing becomes. Percussion in the third stage yields less and less dulness until the exudate has been absorbed. Auscultation in third stage finds the bronchial breathing disappearing. Again the crepitant râle may be heard, then the subcrepitant as the exudate becomes more liquefied, and finally the vesicular murmur returns. Should purulent infiltration set in the temperature remains high, the pulse is frequent and weak, prostration is marked, bronchial breathing continues; also dulness on percussion. A discovery of minute gurgling râles may complete the diagnosis. Termination in gangrene is known by great prostration or collapse. We look for the gangrene odor.

Chronic interstitial pneumonia or pulmonary tuberculosis in the horse is rarely seen, but may occur in old horses. It may arise from the extension of an ordinary pneumonia. It is accompanied by formation of tubercle or caseous material.

Catarrhal pneumonia is always secondary, due to the extension of the inflammation from a previous bronchitis. Acute bronchitis is an inflammatory condition of a part or the whole of the mucous membrane of the large bronchial tubes. In the first stage we find congestion of the mucous membrane. It becomes dry and swells. The tubes become constricted and diminished in calibre. In the second stage we have a catarrhal exudate. The glands are enlarged, mucous membrane swollen and tubes constricted. Auscultation in the first stage finds the sonorous râle. It is heard on both sides, both on inspiration and expiration. The sound is best heard over middle third between sixth and twelfth ribs. The sonorous sound is due to the dryness of the tubes in connection with their diminished calibre. In the second stage when the exudate is thrown out we get a mucous râle both on inspiration and expiration. The sound comes and goes. The secretion may or may not be expectorated. We may not always hear the mucous râle. Percussion in the first stage usually gives an increased resonance, which in fat horses it is almost impossible to distinguish. In the second stage the change in resonance on percussion is slight.

*In capillary bronchitis* percussion yields increased resonance on both sides in both stages unless in old animals. There is on auscultation a mucous râle and hissing sound. In second stage we find a submucous râle. Old animals may not be able to cough up the mucus which may block some of the tubes.

*Asthma.* This morbid condition is a neurosis. It gives rise to paroxysms of dyspœna at recurring intervals. It is probably due to constriction of the circular fibres of the bronchial tubes which causes a wheezy breathing while the paroxysm continues.

*Pulmonary Emphysema.* This is a condition characterized by loss of elastic power in the air-cells. The walls become stretched and contain more than the natural amount of air. There are two varieties, the vesicular and the interstitial. In the former the excess of air is in the air-cells; in the latter it is in the connective-tissue between the cells. The tissues may become ruptured and the cells come together, forming large bladders. Inspection notes that the chest-walls may be enlarged. The abdominal muscles are brought into use in proportion as the disease has advanced. The expiratory movement is much prolonged and consists of three parts: a movement; a pause; then another movement. Percussion gives increased resonance over portions of the chest. Auscultation finds a variety of sounds: In a general way we may say the sounds are of a crepitant, sibilant, and sonorous character.

*Broken Wind.* This name is still applied to a condition of difficult respiration closely resembling that of pulmonary emphysema, a spasmodic breathing with a short inspiration and a prolonged and double expiratory movement. The malady is of nervous origin and asthmatic in character, lacking the recurring paroxysms, but exhibiting a more or less continuous dyspœna. In this disease structural changes have taken place in the circular fibres of the smaller bronchial tubes. This muscular fibre is in a state of constant contraction, having become so from continuous irritation of the nerve-filament supplying the muscle. This irritation is of gastric origin. The integrity of the alveolar passages and air-cells is impaired in time through innutrition; degeneration follows, with production of pulmonary emphysema.

*Pleurisy.* This is an acute inflammation of the serous membrane lining the chest-cavity, forming the mediastinum and covering the lungs. It may be partial or general. The first pathological character is congestion of the bloodvessels of the pleura and the subserous connective-tissue. At first the membrane is dry. This is followed by a fibro-serous exudate. The pleura becomes œdematous in patches. It becomes thickened, loses its polished appearance, becoming dull and rough. The fibrinous lymph of the exudate forms membranes or deposits on the pleura. The serous portion of the exudate falls to the bottom of the cavity. The false membrane may build up and form thick fibrous structures. These may be cartilaginous or osseous in structure. Such structures are rare and are not due to a simple exudate, but a proliferation of the cell-elements of the pleura. The liquid exudate is absorbed by the lymphatics. Inspection in the first stage of pleurisy notes that the respirations are short and quick, especially the inspiratory act. Evidently the act of respiration causes pain. The abdominal type of breathing is increased. The chest is enlarged in the latter stage and may be unequal. Auscultation in the first stage gives the frictionmurmur, a sound similar to that obtained by rubbing together the palms of the hands. It is caused by the now swollen membranes coming in contact with each other in the movements of the lung. Auscultation in the second stage denotes loss of respiratory murmur in the inferior third of the chest and upward in proportion to the depth of the effusion. Percussion in the first stage yields a slightly diminished pulmonary resonance, but dulness increases as the disease advances. Percussion over the effusion yields a flat sound. Above the fluid-line there may be a semi-tympanitic resonance which will be referred to elsewhere.

*Œdema of the Lungs.* This is a condition characterized by an effusion, serous in character, into the air-cells and possibly into the surrounding connective-tissue. It is a bilateral condition. It is a sequel of some debilitating disease. It may appear with congestion of the lungs. It happens suddenly. Inspection notes difficult but feeble respiration. There is no elevation of temperature. Percussion yields dulness. Auscultation may elicit moist râles.

*Pulmonary Congestion.* This condition is caused by a sudden influx of an increased quantity of blood into the pulmonary vessels. Inspection reveals dyspnœa and frothy expectoration tinged with blood. The animal may appear excited or depressed. On auscultation the cardiac sounds are increased. The respiratory murmur is diminished and bronchial breathing increased. On percussion there is loss of pulmonary resonance on both sides of the chest. It is a bilateral malady. Autopsy reveals the lungs deep dyed and crepitant.

Differential Diagnosis: The differential diagnosis of these various diseases of the lungs has made itself apparent in what has been already said of them. It only remains to restate the distinctions in a concise manner.

Croupous pneumonia in the first stage is distinguished from bronchitis in the same stage by the former having a crepitant and the latter a sonorous râle. In bronchitis there is no consolidation of lung-tissue as in pneumonia. It is a bilateral disease. It yields mucous râles in the second stage and increased resonance on percussion. All these are the opposite in pneumonia. Croupous pneumonia is known from capillary bronchitis by about the same signs as bronchitis. In capillary bronchitis we have in the first stage a hissing sound, the sibilant râle. Croupous pneumonia is known from pleurisy by the friction-murmur of the latter in the first stage. In the second stage by flatness on percussion. The upward limit of this loss of resonance is bounded by a horizontal line. Pleurisy with effusion is a bilateral disease in the horse. In pleurisy we have increased abdominal breathing and abdominal furrow.

Pneumonia is distinguished from asthma by one being febrile and the other a non-febrile disorder. Pneumonia is regular in its course. Asthma comes in paroxysms. Asthma is characterized by a wheezy breathing performed with much labor during the paroxysm. In pneumonia the breathing is panting in character.

A paroxysm of asthma is distinguished from an attack of congestion of the lungs by the wheezy breathing of the former. In asthma, on percussion pulmonary resonance is greatly exaggerated and auscultation yields an increase of the respiratory murmur and sibilant râles. These are of the opposite character in congestion of the lungs. There is little or no fever in asthma, and in a severe attack the animals cough very frequently. Asthma may be confounded with bronchitis in the first stage, but the high temperature of bronchitis and the congestion of the Schneiderian membrane and conjunctiva will establish the difference.

Congestion of the lungs may be distinguished from the congestive stage of pneumonia by the latter having very high fever and being ushered in by a chill. Pneumothorax, or air in the chest-cavity, may be distinguished by well-marked tympanitic resonance on percussion. It must not be confounded with the condition that sometimes exists in pleurisy or pneumonia that gives rise to a diminished tympanitic resonance. In pneumonia a tympanitic sound is transmitted through a portion of consolidated lung on heavy percussion in some rare cases. It is supposed to be caused by the vibration of air in a large bronchus or in a cavity. Again, in pleurisy with much effusion we find a tympanitic resonance above the fluid-line. It may be caused by relaxation of the air-sacs and diminished tension of the lung-tissue.

Finally, there is no royal road to physical diagnosis. Its problems must be worked out for one's self. Originality is not claimed for this paper. The ideas are modified from personal experience, observation, and mode of expression. My object has not been so much to teach as to refresh you in the matters which, above all others, combine to make the successful diagnostician.

